# The Contribution of Fortified Ready-to-Eat Cereal to Vitamin and Mineral Intake in the U.S. Population, NHANES 2007–2010

**DOI:** 10.3390/nu7063949

**Published:** 2015-05-25

**Authors:** Victor L. Fulgoni, Rita B. Buckley

**Affiliations:** 1 Nutrition Impact, LLC, 9725D Drive North, Battle Creek, MI 49014, USA; 2 Buckley/Swartz, 300 Lynn Shore Drive, #603, Lynn, MA 01902, USA; E-Mail: rbuckley@buckley-swartz.com

**Keywords:** ready-to-eat cereal (RTEC), fortification, micronutrient intakes, Estimated Average Requirements (EAR), Tolerable Upper Intake Levels (UL), adequate intake (AI)

## Abstract

Micronutrients play a pivotal role in achieving and maintaining optimum health across all life stages. Much of the U.S. population fails to meet Estimated Average Requirements (EARs) for key nutrients. This analysis aims to assess the contribution of fortified ready-to-eat cereals (RTEC) to micronutrient intake for U.S. residents aged 2–18, 19–99, and 2–99 years of age according to National Health and Nutrition Examination Survey (NHANES) 2007–2010 data. We used the National Cancer Institute (NCI) method to assess usual intake of 21 micronutrients and the percentage of the population under EARs and above Tolerable Upper Intake Levels (UL). Without fortification of RTECs, the percentage of those aged 2–18 years that were below EARs increased by 155%, 163%, 113%, and 35% for niacin, iron, thiamin, and vitamin A, respectively. For vitamins B6 and zinc, the respective numbers were 118% and 60%. Adults aged 19–99 and 2–99 had lower percentages but similar outcomes. RTECs are associated with improved nutrient adequacy and do not widely affect prevalence above the UL. The data indicate that large proportions of the population fail to achieve micronutrient sufficiency without fortification, and that its use can help Americans reach national nutrient intake goals.

## 1. Introduction

Micronutrients play a pivotal role in achieving and maintaining optimal health across all life stages [1]. Vitamins are essential nutrients for many body functions, and are particularly important during growth as well as for certain vulnerable groups, such as pregnant women, young children, and the elderly. In addition to increasing nutrient intakes within the population, fortification has an impact on consumer purchasing decisions that can ultimately affect health and well-being [2].

Despite the availability of diverse foods, large-scale population-based dietary intake surveys, such as NHANES, indicate a gap between vitamin intakes and estimated average requirements for a significant proportion of the population; for example, more than 75% of the U.S. population does not get enough vitamin E [3]. A high percentage of all children/adolescents have inadequate intakes of numerous micronutrients, with the greatest inadequacy among older girls [4]. Many Americans would not achieve recommended micronutrient intakes without fortification of the food supply [2].

According to the Dietary Guidelines for Americans 2010 [5], consumption of vegetables, fruits, whole grains, milk and milk products, and seafood is lower than recommended. This makes some micronutrients—potassium, dietary fiber, calcium, and vitamin D—low enough to be a public health issue. Other vitamins of concern include iron, folate, and vitamin B12 [5].

Shortfalls in nutrition intakes could result from changing lifestyles, with increased food choices that have low micronutrient densities. These products have an impact on the quality of an individual’s daily diet, and thus, nutrition status [3].

Diet and physical inactivity are the most important contributors to the epidemic of overweight and obesity among men, women, and children in all segments of our society [5]. Fortified ready-to-eat cereals (RTEC) with lower calories appear to be an effective tool for addressing nutritional deficiencies among populations [6]; for instance, encouraging RTEC consumption by pregnant women is a simple, safe, and inexpensive intervention that could help optimize nutrient intake for successful placental and fetal development [7].

Fortified RTECs remain a major contributor of micronutrients within the U.S. diet. Enriched and/or fortified foods provide a large proportion of the intakes of vitamins A, C and D, as well as thiamin, iron, and folate, although some of these nutrients are still below the EARs for a significant portion of the population [8]. Trends observed in the types of fortification in RTECs demonstrate positive changes in nutrient composition that may have an important impact on public health [9].

It is also important to consider fortification in the context of potential micronutrient overexposure. For example, excessive niacin intake can lead to potential skin reactions; too much vitamin A can lead to liver damage; and high levels of zinc can inhibit immune function [10]. Whether intake of some nutrients in fortified foods will result in total consumption above the UL depends, in part, on how the upper intake thresholds are estimated and how the target population is assessed [2].

The aim of this study was to determine the impact of RTEC fortification on micronutrient sufficiency in U.S. youths aged 2–18 and adults 19–99 and 2–99 years of age.

## 2. Subjects and Methods

### 2.1. Study Design

We performed a secondary analysis of data from the National Health and Nutrition Examination Survey (NHANES) 2007–2010. NHANES is a continuous cross-sectional survey on the health and nutrition status of a nationally representative sample of the civilian, non-institutionalized population of the United States [11]. The database is a publicly available resource for use by researchers throughout the world. No permission or IRB approval is needed to access NHANES data.

NHANES uses a complex, stratified, multistage probability cluster sampling design to collect demographic, socioeconomic, dietary, and general health data. Survey participants undergo a comprehensive health examination in a mobile examination center (MEC). Trained interviewers collect dietary data via in-person 24 h dietary recalls using USDA automated multi-pass methods during the MEC examination [12]. A second 24 h dietary recall is conducted by telephone 3–10 days after the first. Survey participants 12 years and older complete the dietary interview on their own. Proxy respondents report for children less than 11 years of age.

For this analysis, we used the 2007–2010 NHANES data set for ages 2–18 (*n* = 6090), 19–99 (*n* = 11,297), and 2–99 years (*n* = 17,387), with exclusions for unreliable data and pregnant or lactating females. We only included dietary records deemed reliable and complete by the USDA Foods Surveys Research Group.

The analysis included 21 different micronutrients—vitamin A, thiamin, riboflavin, niacin, total choline, vitamin B6, vitamin B12, folate (DFE), vitamin C, vitamin D, vitamin E (as alpha-tocopherol), vitamin K, calcium, phosphorus, magnesium, iron, zinc, copper, selenium, sodium, and potassium. We estimated amounts consumed from RTECs with fortification, and then used modeling that removed all 21 nutrients from RTECs to estimate intakes without fortification. Given that there is no way to estimate inherent nutrients in all RTEC, we assumed that all nutrients added were from fortification. Dietary supplements and medicines were excluded. All micronutrients are shown in Table 1, Table 2, Table 3 and Table 4; only statistically significant (*p* < 0.05) results are included in the Results and Discussion sections.

Cereal consumers were defined as individuals who had eaten some RTEC on the first NHANES 24 h dietary recall sample day. Dietary Reference Intakes (DRIs) for populations came from the U.S. Food and Nutrition Board, Institute of Medicine, and the National Academies of Science.

### 2.2. Statistical Analyses

Intake was determined on daily and usual bases with and without fortification calculated using the National Cancer Institute (NCI) method [13], as were percentiles of intakes, and when applicable, percent of the population below the EAR, AI, and above the UL for the 21 nutrients used in the analysis. The NCI-supplied SAS programs Mixtran v1.1 and Distrib v1.1 were respectively used to generate parameter effects after covariate adjustment and estimate the distribution of usual intake via the Monte Carlo method [14]. The covariates used in the NCI usual intake estimation were sequence coded by day (weekend, Friday through Sunday) or (weekday, Monday through Thursday) and DRI age groups (2–18, 19–99 and 2–99 years).

We used the balanced repeated replication (BRR) method with a non-response adjustment for variance estimates, standard errors, and confidence intervals for usual intake means and percentiles. SAS software (version 9.3, SAS Institute, Cary, NC, USA) was used to perform all analyses. We used dietary sample weights to account for differential non-response, adjust for oversampling of some groups, and account for the complex sample design of NHANES [15]. We used a Z-statistic to determine differences in population groups, and conducted food source analyses and sources of nutrients in the diets of children and adults, respectively. The level of significance was set at *p* < 0.05.

RTECs are major dietary sources of micronutrients. Thus, we created separate categories for 21 nutrients in the analyses using data from the first NHANES 24 h recall. We determined the mean and standard error (SE) of nutrient intakes contributed from the total diets and from each food group with PROC DESCRIPT of SUDAAN (version 11.0; RTI International, Research Triangle Park, NC, USA).

## 3. Results

### 3.1. Estimated Average Requirements (EAR) and Contribution of RTEC Fortification

Among those 2–18 years of age with intake below EAR, significant (*p* < 0.05) nutrient increases resulting from RTEC fortification ranged from 3.3% for vitamin D (D2 + D3) to 161.5% for folate, DFE. Beside folate and vitamin D, there were significant increases in iron; thiamin (vitamin B1); riboflavin (vitamin B2); vitamin A, RAE; vitamin B6; vitamin E (alpha-tocopherol); niacin (*p* = 0.01); and zinc (Table 1).

**Table 1:** Percentage of the U.S. population below EAR or AI ^a^ with and without micronutrient fortification, ages 2–18 (*n* = 6090).

Micronutrient	With Fortification	No Fortification	% Increase *
Calcium (mg)	45.32 ± 1.05	47.58 ± 1.03	5.0
Copper	3.96 ± 0.48	4.68 ± 1.03	18.1
Folate, DFE (mcg) ^c^	4.42 ± 0.46	11.56 ± 1.03	161.5 *
Iron (mg) ^c^	2.43 ± 0.25	6.40 ± 0.47	16.3 *
Magnesium (mg)	35.35 ± 0.96	37.60 ± 0.96	6.3
Niacin (mg) ^c^	0.60 ± 0.16	1.53 ± 0.34	155.0 *
Phosphorus (mg)	16.29 ± 0.95	17.67 ± 0.94	8.4
Potassium	98.22 ± 0.20	98.4 7± 0.16	0.2
Vitamin B2 (mg) ^c^	1.38 ± 0.20	2.31 ± 0.31	67.3 *
Selenium (mcg)	0.19 ± 0.07	0.23 ± 0.09	21.0
Sodium (mg) ^a^	0.53 ± 0.14	0.72 ± 0.18	35.8
Thiamin (vitamin B1, mg) ^c^	2.15 ± 0.38	4.58 ± 0.64	113.0 *
Total choline (mg) ^a^	77.99 ± 0.83	78.65 ± 0.81	0.85
Vitamin A, RAE (mcg) ^c^	24.90 ± 0.98	33.70 ± 0.88	35.3 *
Vitamin B12 (mcg) ^c^	1.51 ± 0.98	2.48 ± 0.49	64.2
Vitamin B6 (mg) ^c^	3.29 ± 0.42	7.18 ± 0.80	118.2 *
Vitamin C (mg)	21.34 ± 0.82	23.77 ± 0.97	11.3
Vitamin D (D2 + D3, mcg)	88.21 ± 0.69	91.14 ± 0.55	3.3 *
Vitamin E ^b^ (mg)	77.77 ± 0.79	80.83 ± 0.77	3.9 *
Vitamin K (mcg) ^a^	58.84 ± 1.27	59.37 ± 0.77	0.9
Zinc (mg) ^c^	8.55 ± 0.89	13.75 ± 1.15	60.8 *

^a^ Percentages below adequate intake (AI) levels where EAR was not available; ^b^ alpha-tocopherol; * *p* < 0.05; ^c^ RTEC contributes 10% or more of the mean usual micronutrient intake.

In the 19–99 year old age group, significant percentage increases for nutrients below EAR ranged from 8.3% for magnesium to 84.8% for folate. Other micronutrient intakes that increased as a result of RTEC fortification were iron; riboflavin (vitamin B2); thiamin (vitamin B1); vitamin A, RAE; vitamin B12; vitamin B6; zinc; niacin; and vitamin E (Table 2).

**Table 2:** Percentage of the U.S. population below EAR or AI ^a^ with and without micronutrient fortification, ages 19–99 (*n* = 11,297).

Micronutrient	With Fortification	No Fortification	% Increase *
Calcium (mg)	42.07 ± 0.70	44.00 ± 0.68	4.5 *
Copper	4.12 ± 0.55	4.93 ± 0.63	19.6
Folate, DFE (mcg) ^c^	10.77 ± 0.61	19.91 ± 0.94	84.7 *
Iron (mg) ^c^	4.58 ± 0.32	6.99 ± 0.36	52.2 *
Magnesium (mg)	55.85 ± 0.78	60.50 ± 0.73	8.3 *
Niacin (mg) ^c^	1.30 ± 0.28	2.34 ± 0.39	80.0 *
Phosphorus (mg)	0.74 ± 0.17	0.85 ± 0.18	14.8
Potassium	97.73 ± 0.29	98.06 ± 0.27	0.3
Riboflavin (vitamin B2, mg)	2.23 ± 0.20	3.04 ± 0.25	36.3 *
Selenium (mcg)	0.39 ± 0.13	0.50 ± 0.16	28.2
Sodium (mg) ^a^	0.25 ± 0.10	0.31 ± 0.12	24.0
Thiamin (vitamin B1, mg) ^c^	5.28 ± 0.63	8.90 ± 0.91	68.5 *
Total choline (mg) ^a^	94.12 ± 0.53	94.32 ± 0.50	0.21
Vitamin A, RAE (mcg) ^c^	46.84 ± 1.15	54.84 ± 1.11	17.0 *
Vitamin B12 (mcg) ^c^	2.75 ± 0.33	4.27 ± 0.61	55.2 *
Vitamin B6 (mg) ^c^	11.18 ± 0.79	18.07 ± 0.99	61.6 *
Vitamin C (mg)	42.02 ± 0.85	44.83 ± 0.86	4.2
Vitamin D (D2 + D3, mcg)	95.71 ± 0.45	96.75 ± 0.37	4.2
Vitamin E ^b^ (mg)	91.18 ± 0.61	93.37 ± 0.60	2.4 *
Vitamin K (mcg) ^a^	68.37 ± 0.99	68.39 ± 0.98	0.03
Zinc (mg) ^c^	12.32 ± 0.72	17.09 ± 0.88	38.7 *

^a^ Percentages below adequate intake (AI) levels where EAR was not available; ^b^ alpha-tocopherol; * *p* < 0.05; ^c^ RTEC contributes 10% or more of the mean usual micronutrient intake.

Significant percentage increases toward EAR in the population aged 2–99 years ranged from 2.7% for vitamin E to 91.9% for folate, DFE. Other nutrients that showed higher intakes with fortification of RTECs were iron; magnesium; riboflavin (vitamin B2); thiamin; vitamin A, RAE; vitamin B6; zinc, calcium; niacin; vitamin B12; vitamin C; and vitamin D (Table 3).

Table 4 shows the contribution of RTEC fortification to micronutrient intake (% increase compared to when fortification was removed) across all age groups.

### 3.2. Percentage of Population with Micronutrient Intake above the UL

Sodium was above the UL in 89.26% ± 0.93% of those in the 2–18 year age group and 89.27% ± 0.79% of those aged 19–99 years. Removing fortification reduced these levels by 2.3% and 1.4%, respectively. Among those aged 2–18 years, the prevalence of potential risk from excessive micronutrient intake (>UL) was <1% for calcium and iron. Risk of overconsumption was 14.73% ± 0.73% for zinc, with a 41.9% decline without fortification, and 2.37% ± 0.22% for selenium, with a 12.6% decline. Among those aged 19–99 years, <1% of the population was above the ULs for calcium and iron. In those aged 2–99 years, 3.58% ± 0.18% of the population was above the UL for zinc, with a 42.1% decline without fortification.

**Table 3:** Percentage of the U.S. population below EAR or AI ^a^ with and without micronutrient fortification, ages 2–99 (*n* = 17,387).

Micronutrient	With Fortification	No Fortification	% Increase *
Calcium (mg)	42.87 ± 0.62	44.87 ± 0.62	4.6 *
Copper	4.12 ± 0.49	4.79 ± 0.54	16.2
Folate, DFE (mcg) ^c^	9.33 ± 0.53	17.91 ± 0.81	91.9 *
Iron (mg) ^c^	4.06 ± 0.28	6.85 ± 0.33	687 *
Magnesium (mg)	50.82 ± 0.57	55.05 ± 0.60	8.3 *
Niacin (mg) ^c^	1.12 ± 0.23	2.18 ± 0.36	94.6 *
Phosphorus (mg)	4.51 ± 0.30	4.96 ± 0.32	9.9
Potassium	97.81 ± 0.27	98.16 ± 0.23	0.36
Riboflavin (vitamin B2, mg)	2.00 ± 0.19	2.88 ± 0.25	44.0 *
Selenium (mcg)	0.34 ± 0.11	0.44 ± 0.13	29.4
Sodium (mg) ^a^	0.30 ± 0.11	0.41 ± 0.13	36.6 *
Thiamin (vitamin B1, mg) ^c^	4.48 ± 0.53	7.83 ± 0.78	74.7 *
Total choline (mg) ^a^	90.19 ± 0.49	90.56 ± 0.46	0.4
Vitamin A, RAE (mcg) ^c^	41.53 ± 0.97	49.74 ± 0.93	19.7 *
Vitamin B12 (mcg) ^c^	2.47 ± 0.29	3.84 ± 0.55	55.4 *
Vitamin B6 (mg) ^c^	9.27 ± 0.68	15.46 ± 0.84	66.7 *
Vitamin C (mg)	37.73 ± 0.66	39.65 ± 0.62	5.0 *
Vitamin D (D2 + D3, mcg)	93.91 ± 0.46	95.42 ± 0.38	1.6 *
Vitamin E ^b^ (mg)	87.90 ± 0.56	90.35 ± 0.53	2.7 *
Vitamin K (mcg) ^a^	66.03 ± 0.84	66.22 ± 0.83	0.2
Zinc (mg) ^c^	11.47 ± 0.69	16.33 ± 0.82	42.3 *

^a^ Percentages below adequate intake (AI) levels where EAR was not available; ^b^ alpha-tocopherol; * *p* < 0.05; ^c^ RTEC contributes 10% or more of the mean usual micronutrient intake.

**Table 4:** Contribution of RTEC fortification to micronutrient intake (% increase) across all age groups.

Micronutrient	Ages 2–18	*p*-Values ^a^	Ages 19–99	*p*-Values ^a^	Ages 2–99	*p*-Values *
	% Increase		% Increase		% Increase	
Calcium	5.0	0.12	4.5	0.04 *	4.6	0.02 *
Copper	18.1	0.31	19.6	0.33	16.2	0.35
Folate, DFE	161.5	0.00 *	84.8	0.00 *	91.9	0.00 *
Iron	163.3	0.00 *	52.6	0.00 *	68.7	0.00 *
Magnesium	6.3	0.09	8.3	0.00 *	8.3	0.00 *
Niacin	155.0	0.01 *	80.0	0.02 *	94.6	0.01 *
Phosphorous	8.4	0.30	14.8	0.63	9.9	0.30
Potassium	0.25	0.32	0.34	0.39	0.36	0.33
Riboflavin (vitamin B2)	67.3	0.01 *	36.3	0.01 *	44.0	0.00 *
Selenium	21.0	0.66	28.2	0.60	29.4	0.57
Sodium	33.8	0.40	24.0	0.69	36.6	0.02 *
Thiamin (vitamin B1)	113.0	0.00 *	68.5	0.00 *	74.7	0.00 *
Total choline	0.85	0.56	0.21	0.77	0.41	0.57
Vitamin A, RAE	35.3	0.00 *	17.0	0.00 *	19.7	0.00 *
Vitamin B12	64.2	0.09	55.2	0.02 *	55.4	0.02 *
Vitamin B6	118.2	0.00 *	61.6	0.00 *	66.7	0.00 *
Vitamin C	11.3	0.05	4.2	0.13	5.0	0.03
Vitamin D	3.3	0.00 *	1.0	0.07	1.6	0.01 *
Vitamin E	3.9	0.00 *	2.4	0.01 *	2.7	0.00 *
Vitamin K	0.90	0.77	0.03	0.99	0.29	0.87
Zinc	60.8	0.00 *	38.7	0.00 *	42.3	0.00 *

* Statistically significant (*p* < 0.05).

### 3.3. Percentage of Individuals below the AI with Fortified RTEC Micronutrients

Analysis of NHANES 2007–2010 data indicates that even with fortification of RTECs, certain nutrients (*i.e.*, potassium, total choline, and vitamin K) are below AI across all age groups. Inadequate intakes of potassium affected approximately 98% of all individuals. The respective figures for total choline below AI for those aged 2–18, 19–99, and 2–99 years were 77.99% ± 0.83%, 94.12% ± 0.53%, and 90.19% ± 0.49%; for vitamin K, they were 58.84% ± 1.27%, 94.12% ± 0.53% and 66.03% ± 0.84%, respectively.

## 4. Discussion

To our knowledge, our observations are the first to model the potential impact of a lack of fortification on overall dietary intake among a nationally representative sample of U.S. youths and adults who consume fortified and nonfortified RTECs. The prevalence of dietary inadequacy (assessed as the proportion of the population with intakes below the EAR) was significantly lower for consumers of fortified RTECs compared with nonfortified RTECs.

The results of this study confirm prior reports of higher mean nutrient intakes from food sources among breakfast consumers and/or those who ate RTECs for breakfast in nationally representative samples of American children and young adults [16,17] as well as Australian boys [18] and adults [19]. Galvin *et al.* [20] found that in Irish adults, fortified RTECs were associated with a more nutrient-dense diet and a reduced risk of dietary inadequacy for calcium, iron, riboflavin, and folate. These outcomes did not increase the risk of excessive intakes of micronutrients [20].

Barr *et al.* [21] demonstrated that among Canadian children and adolescents, the prevalence of nutrition inadequacy for vitamin D, calcium, iron, and magnesium was lowest in consumers of RTEC breakfasts compared with those who skipped breakfast or ate other types of breakfasts. In all groups, the potential risk of excessive nutrient intakes was low.

Food fortification is a proven and effective tool for tackling nutritional deficiencies among populations, especially among ‘emergent deficiencies’ that were not previously considered a problem [6].

In the U.S., current micronutrients of public health concern include potassium, calcium, and vitamin D. For specific population groups (e.g., those 50 years and older and women of childbearing age), intakes of iron, folate, and vitamin B12 are also of concern [5]. Several of the nutrients enhanced by RTEC consumption iron, folate, vitamin B12, and vitamin D. RTEC fortification increased the consumption of these nutrients across all age groups, particularly among those aged 2–18 years (Table 4). However, it fell short of AI for potassium, total choline, and vitamin K.

Overall, fortification improved nutrient adequacy and had scant impact on the prevalence of intakes with slightly higher proportions above the ULs. Intakes in excess of the UL were not associated with the addition of these micronutrients to RTECs. In addition, the proportion of individuals with intakes exceeding the UL remained largely unchanged when the added micronutrients were excluded from the intake estimate [20]. This indicates that consumption above the ULs was not driven by fortification.

Although the potential for nutrient overconsumption cannot be disregarded, particularly in the case of zinc in the 2–18 year and 2–99 year age groups, the benefits of fortified RTECs on reducing nutrient inadequacy appear to outweigh the potential risks for adverse effects of excess intake [22]. This study found that fortified RTEC was associated with better nutrient adequacy and did not meaningfully affect prevalence above UL for all micronutrients other than zinc.

### Strengths and Limitations

The strengths of this report include a large, nationally representative sample with validated data collection, and the use of sophisticated, readily available software to estimate intake distributions (*i.e.*, the NCI method). Another includes the use of advanced modeling techniques to estimate consumption of RTECs without fortification among the various age groups.

Limitations include the assessment of dietary nutrient adequacy and excess based on food sources alone, without the potential contribution of supplements. NHANES data are also cross-sectional (points in time) and rely on different subjects each year. In addition, we assumed that all nutrients examined in RTEC were from fortification, but there may have been small amounts of some nutrients still in RTEC after processing; thus, we may have slightly overestimated the impact of fortification removal.

## 5. Conclusions

This study supports previous reports that demonstrate the positive impact of fortified cereals on micronutrient intakes in the diets of adults [22] and children [23], with relatively small risk for adverse effects from excessive intake [1,22]. It shows that RTEC fortification is a cost-effective way to improve vitamin and mineral intake and ensure a healthy and productive life for many at-risk individuals in the U.S. [3].
